# Supplementary material for: Anoxic chlorophyll maximum enhances local organic matter remineralization and nitrogen loss in Lake Tanganyika
Source: Nat Commun. 2021 Feb 5;12:830. doi: 10.1038/s41467-021-21115-5 (PMC7864930; doi:10.1038/s41467-021-21115-5)
Supplement: Supplementary file 1 — Supplementary Information [file 41467_2021_21115_MOESM1_ESM.pdf]

Supplementary information for Anoxic chlorophyll maximum enhances local organic matter remineralization and nitrogen loss in Lake Tanganyika by Callbeck et al.,

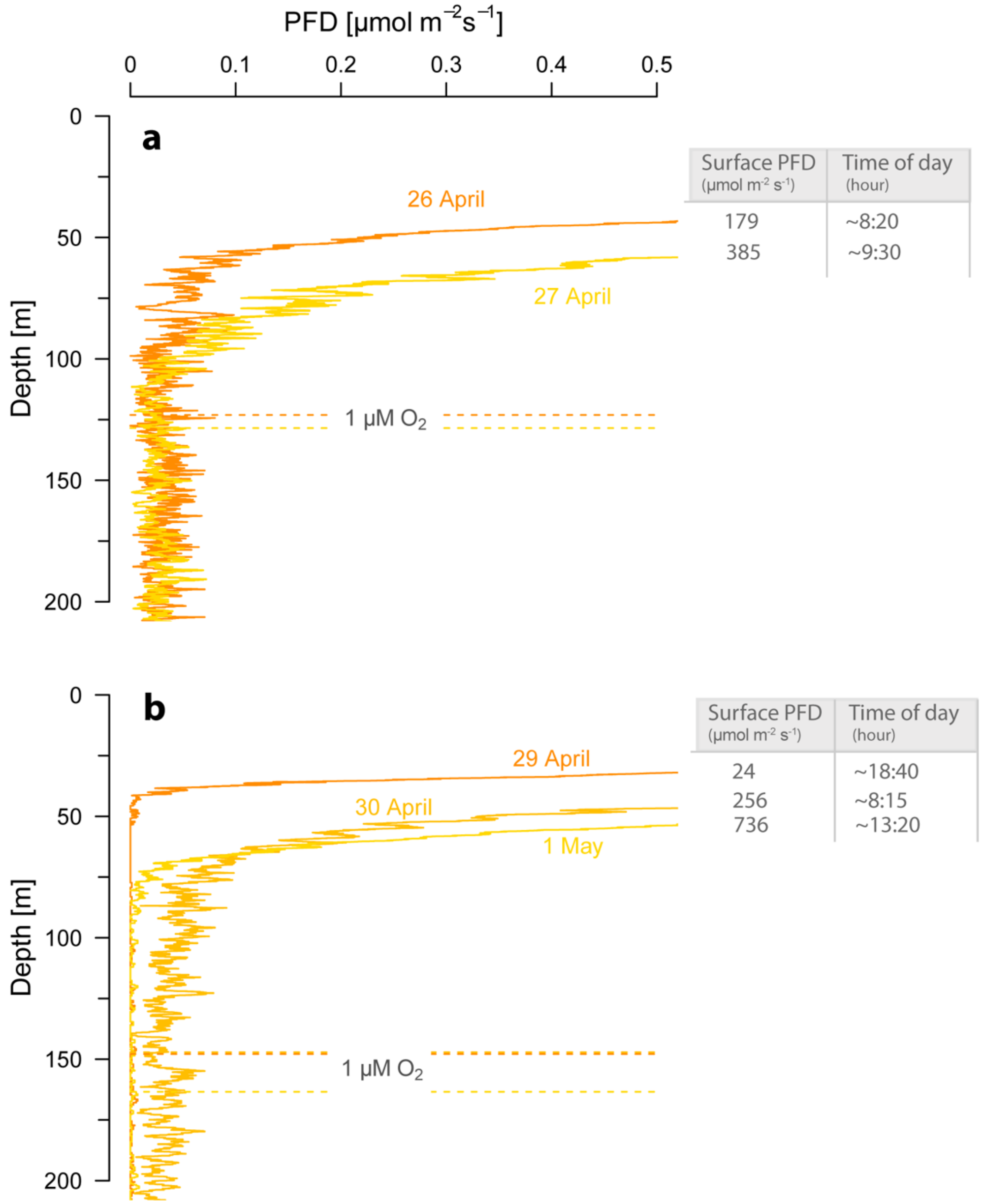

Supplementary Figure S1: Vertical light irradiance profiles for Lake Tanganyika. Data shown is from the April-May 2019 campaign. (**a, b**) The photosynthetically active radiation is expressed as the photon flux density (PFD) for stations 2 and 7, which are indicated in the top and bottom panels, respectively. The corresponding surface light irradiance values, and the time of day when the measurements were performed are indicated in the adjacent table. Note that the small secondary increase in the irradiance value at the anoxic chlorophyll maximum (145 m; mainly observed with the April 26 profile at station 2), is likely attributed to changes in the surface irradiance, and possibly to minor reflectance induced by particles and phototrophic cells at this depth (Fig. 2a, c).

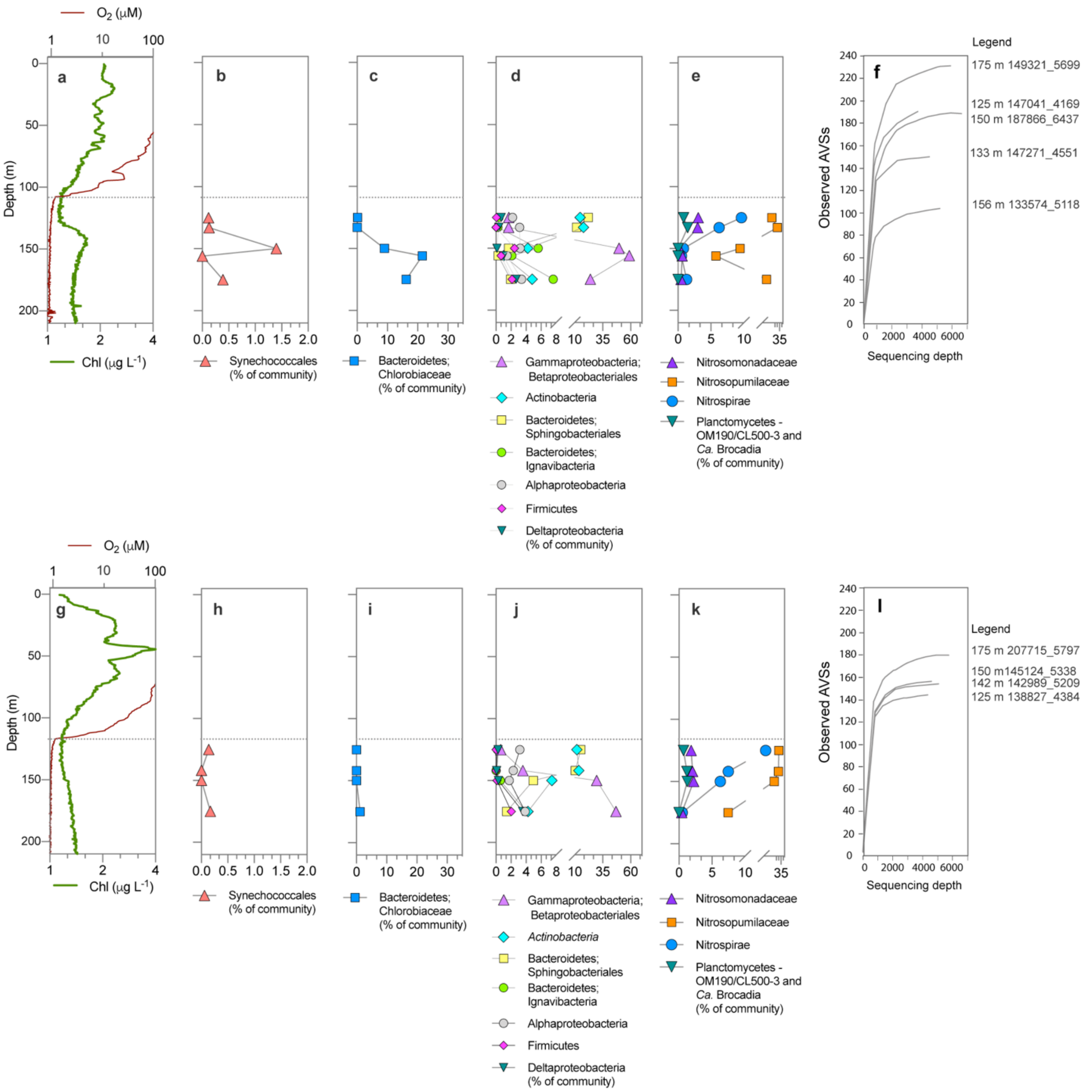

Supplementary Figure S2: Recovered 16S rRNA gene abundances for key taxa identified in Lake Tanganyika. Data shown is from the April-May 2018 campaign. Stations 2 and 7 are indicated in the top (a-f) and bottom (g-l) panels, respectively. The oxygen deficient zone is delimited at an O_2 cutoff of 1 µM indicated by the dotted line. The percent community is based on the total number of amplicon sequence variants (AVSs). The values shown beside the rarefaction curves, in panels f and l, indicate the sample depth, the number of raw reads and the number of filtered ASVs per sample, respectively. Note that the x-axes related to oxygen and chlorophyll profiles are not shown on linear scales.

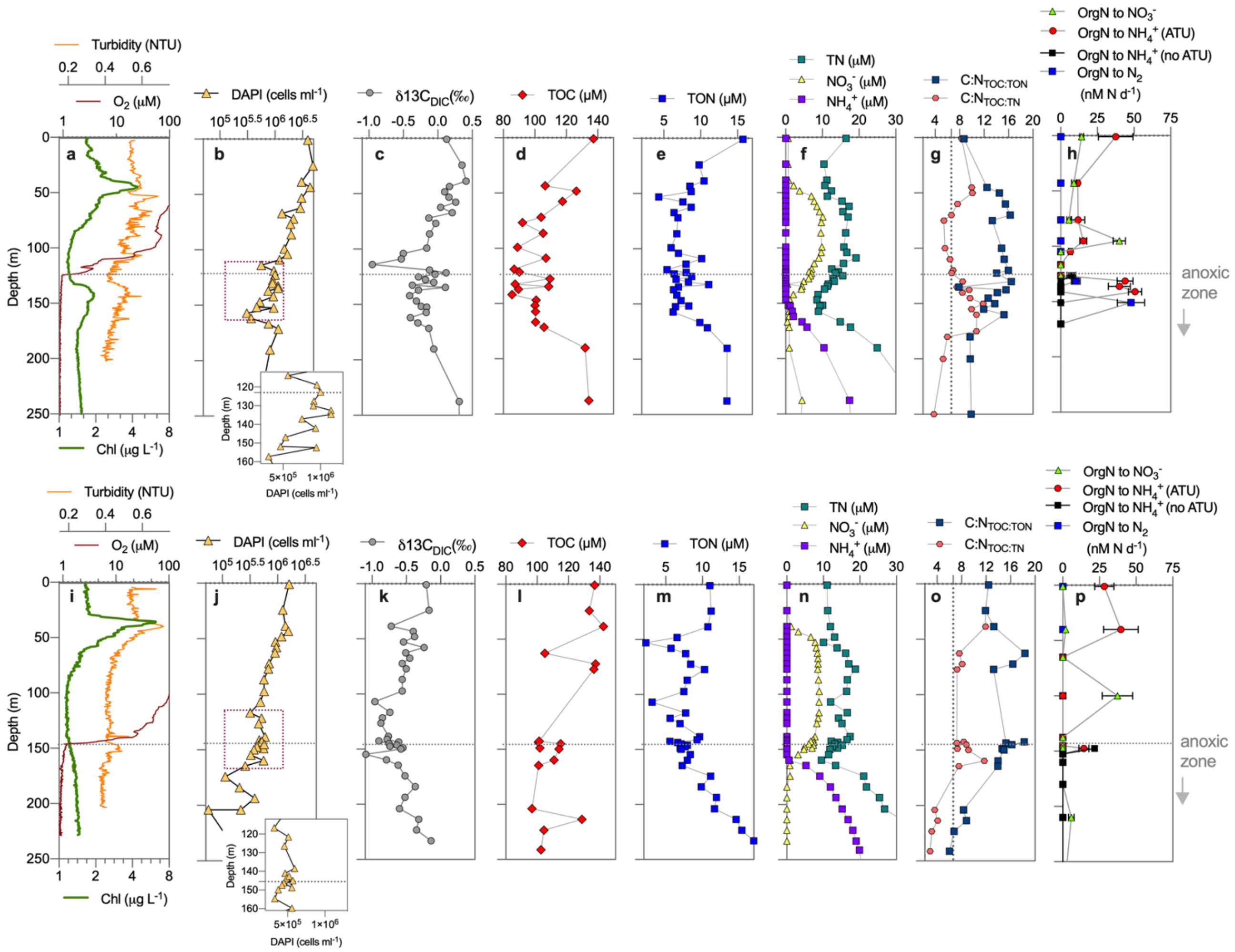

Supplementary Figure S3: Vertical distribution of bacterial cell counts, total organic matter, and organic nitrogen remineralization rates in Lake Tanganyika. Data shown is from the April-May 2019 expedition. Stations 2 and 7 are indicated in the top (a-h) and bottom (i-p) panels, respectively. The oxygen deficient zone is delimited at an O_2 cutoff of 1 μM indicated by the dotted line. Note that the x-axes related to oxygen and chlorophyll profiles are not shown on linear scales. In panels d and l, anomalously high TOC concentrations (>2000 μM) were measured in 13 of 36 samples at station 2 and 16 of 33 samples at station 7. In the same samples, however, TN values were normal. We therefore attributed the anomalously high TOC values (not shown in panels d and l) to an unknown instrument error associated with the TOC analysis. In panels g an o, the dotted vertical line represents the Redfield C:N ratio of 6.6. Rates of organic nitrogen remineralization from the $^{13}C/^{15}N$-algal additions, to ammonium, nitrate and N_2 are indicated in panels h and p; error bars represent the standard error. Abbreviations are as follows: TOC, total organic carbon; DIC, dissolved inorganic carbon; TON, total organic nitrogen; ATU, allylthiourea (nitrification inhibitor).

Supplementary Table 1: Summary of PCR primers used in this study.

Target	Primer name	Oligonucleotide sequence (5'-3')	Reference
Prokaryotes	341F	CCTAYGGGRBGCASCAG	[a](Yu et al. 2005)
	806R	GGACTACNNGGGTATCTAAT	[a](Yu et al. 2005)

[a] Primer set is also described by the Novogene sequence center (https://en.novogene.com/services/research-services/metagenomics/16s-18s-its-amplicon-metagenomic-sequencing/)

Reference

Yu, Y., C. Lee, J. Kim, and S. Hwang. 2005. Group-specific primer and probe sets to detect methanogenic communities using quantitative real-time polymerase chain reaction. Biotechnol. Bioeng. **89**: 670–679. doi:https://doi.org/10.1002/bit.20347
